# Right to health and not to be harmed: a definition from Evidence-Based Medicine

**DOI:** 10.1590/S1516-31802010000500001

**Published:** 2010-09-02

**Affiliations:** I MD, PhD. Titular professor and Head of the Discipline of Emergency Medicine and Evidence-Based Medicine of Universidade Federal de São Paulo — Escola Paulista de Medicina (Unifesp-EPM). Director of the Brazilian Cochrane Center and Scientific Director of Associação Paulista de Medicina (APM). E-mail: atallahmbe@uol.com.br

The Brazilian Constitution states in its article 196 that *"Health is everyone’s right and the State’s duty, and is ensured through social and economic policies that aim to reduce the risk of disease and other health hazards and to enable universal and egalitarian access to actions and services for health promotion, protection and recovery*". Most people, through natural bias of their supposed interest, generally only read and cite the first part: health as the citizen’s right and the state’s duty, while forgetting that, as pronounced in article 196, the State also has a duty to avoid health hazards.^1^

Hence, in fulfilling the duty to provide healthcare, it has to be known whether this act will result in more benefit than harm for citizens. The principle of the Hippocratic Art has already guided all of Medicine for millennia: "*Primum non nocere*" (Firstly, do no harm).

In Hippocrates’ treatise "Of the Epidemics", he wrote: "*Practice two things in dealing with diseases: assist and do not harm the patient*". Thus, in caring for health, which has been defined by the World Health Organization (WHO) as *a complete state of physical, mental and social wellbeing*, and not just as *absence of disease*, it is necessary to clarify the effectiveness, efficiency and, especially, the safety of each healthcare decision.^2,3^

In other words, provision of treatment for which the effectiveness and safety have not been adequately studied goes against the Constitution, since such provision may promote health hazards and can often reduce the universal access to treatments that are effective and safe.

The Hippocratic Oath lays down obligations regarding doing no harm and providing benefits: "*I will use my power to help patients, to the best of my ability and judgment; I will refrain from ways of causing harm or deceiving anyone through my power.*"

Since power and economic and financial interests greatly blunt visionary notions, it is of fundamental importance that the right to health that the Constitution refers to should really be based on scientific evidence that at least reduces the likelihood of harm to individuals and to the population.

In this manner, it is clear that the right to health established by the Brazilian Constitution in its article 196 presupposes that health hazards should be prevented. Therefore, not only should the right to health be based on evidence of treatment efficiency and safety, but also the so-called judicialization of medicine should be well received, provided that the decisions made are explained in terms of the best scientific evidence that exists. For this, it is necessary to map out the knowledge in relation to each decision-making process.

At this point, I ask for readers’ forbearance while I go back to WHO’s definition of health, which is that health is a state of complete physical, mental and social wellbeing. Although this definition seems to be very extensive, it is little applied in reality.

I take the view that there is sufficient evidence to say that the right to health begins during the gestational period. It is known that pollution, alcohol and smoking cause harm to babies. We have the right to sleep in silence, and hence there is a need for laws and drastic actions to avoid noise during the night. We have the right to breath air that is less polluted, given that there are large quantities of evidence regarding the health hazards caused by pollution. We have the right to come and go safely and to take exercise in the open air.

We and our children have the right to consume water and food that do not carry substances that are harmful to our health. We have the right not to be plagued by advertising. Pregnant women bring new inhabitants to the country and, for this reason, they have the right to receive transportation and shelter in keeping with maternity and the right to expect that their babies are welcomed with tenderness, security and comfort. In other words, we need to give a good and respectful reception to the innocents who arrive in the world, so that their health is not subject to hazards. And many other rights.

We have the duty to construct a better educated, better informed and healthier society and to be responsible for our acts and for the society within which we live.
